# Family structure, family affluence and adolescents’ subjective well-being: the mediating role of basic psychological need satisfaction

**DOI:** 10.3389/fpsyg.2026.1958295

**Published:** 2026-10-29

**Authors:** Tamara Gordeeva, Oleg Sychev, Olga Kazantseva, Elena Dvorskaya

**Affiliations:** 1 Department of Educational Psychology and Pedagogy, Faculty of Psychology, Lomonosov Moscow State University, Moscow, Russia; 2 Administration of the Governor and the Government of Altai Krai, Barnaul, Russia

**Keywords:** adolescents, basic psychological need satisfaction, family structure, perceived family affluence, residential care, self-determination theory, subjective well-being

## Abstract

**Introduction:**

Adolescents’ subjective well-being has been widely studied across different family structures. However, little is known about the role of basic psychological needs in the subjective well-being of adolescents living in different family structures or in residential care. The present study addresses this gap.

**Methods:**

The sample comprised 5,295 adolescents aged 10 -16 years (*M*_age_ =13.20, SD=1.50) living in intact families (65%), single-parent families (21%), stepparent families (12%), or residential care (2%). Participants completed self-report measures of basic psychological need satisfaction at home, subjective well-being (affective and cognitive components), and perceived family affluence.

**Results:**

Structural equation modeling showed significant indirect associations between family structure and adolescents’ subjective well-being through basic psychological need satisfaction at home, including a sequential indirect association through perceived family affluence and basic psychological need satisfaction. Living in residential care was also indirectly associated with adolescents’ subjective well-being through basic psychological need satisfaction at home, whereas the direct association was not statistically significant. The results show that adolescents living in intact families reported the highest level of basic psychological need satisfaction at home, followed by those in single-parent and stepparent families, while adolescents in residential care reported the lowest level.

**Discussion:**

The findings provide evidence that the association between family and care contexts and adolescents’ subjective well-being is statistically mediated by basic psychological need satisfaction at home. The results are discussed in light of the value of parental and caregiver support for adolescents’ basic psychological needs in different family types and care.

## 1 Introduction

Adolescents’ subjective well-being is an important area of psychological research because it is associated with a range of outcomes extending into adulthood, including physical and mental health (Long et al., 2024; Herke et al., 2026), social relationships and stronger friendships (Kansky et al., 2016), academic achievement (Cárdenas et al., 2022; Gordeeva et al., 2026a), and later career and financial well-being (Diener and Ryan, 2009; De Neve and Oswald, 2012; Kansky et al., 2016). These findings point to the importance of examining characteristics of adolescents’ social environments that are associated with their subjective well-being. Among these characteristics are family structure and care context, which reflect the configuration of parental and caregiving relationships in which adolescents grow up and develop.

An increasing number of children and adolescents live in single-parent and stepparent families (Gurko, 2020; Jensen and Sanner, 2021). Similar trends are observed in Russia, where single-mother families are particularly prevalent (Selivanova and Korobkova, 2024). Research findings indicate that children and adolescents growing up in family structures other than intact families, as well as outside family settings, tend to report lower subjective well-being and life satisfaction (Jensen and Sanner, 2021; Dolgikh, 2025; Semya et al., 2025). The psychological mechanisms underlying the lower well-being and life satisfaction of children and adolescents from different family structures and residential care remain under investigation, but findings are complex and inconsistent. The present study examines adolescents’ basic psychological need satisfaction at home as a potential factor linking residence in different family structures or residential care to their subjective well-being.

### 1.1 Family structure and adolescents’ subjective well-being

Family structure is a widely studied and readily observable family characteristic that makes a small but significant contribution to adolescent well-being. Despite the wide range of family structures identified in contemporary research (Jensen and Sanner, 2021; Ganong and Sanner, 2023), the most prevalent family structures worldwide are intact families, single-parent families, most commonly single-mother families, and stepparent families (Jensen and Sanner, 2021; Ganong and Sanner, 2023). The present study focuses on these family structures, as well as on adolescents living outside family settings in residential care.

A substantial body of research has examined the association between family structure and adolescent well-being, yielding both consistent and divergent findings. The most consistent evidence comes from comparisons between adolescents from intact families and those from non-intact families. In particular, adolescents from single-parent families report lower well-being and life satisfaction (Biblarz and Gottainer, 2000; Bjarnason et al., 2012; Dinisman et al., 2017; Schütz et al., 2019; Herke et al., 2020), higher levels of ill-being, including depression, anxiety, perceived stress, and suicidal ideation (Park and Lee, 2020), and higher levels of internalizing and externalizing symptoms than adolescents from intact families (Herke et al., 2020; Kuruczova et al., 2020; Grüning Parache et al., 2024), including long-term consequences for well-being (Richter and Lemola, 2017). Adolescents from stepparent families also generally report lower life satisfaction (Schütz et al., 2019; Herke et al., 2020) and higher levels of internalizing and externalizing symptoms than their peers from intact families (Herke et al., 2020; Kuruczova et al., 2020; Grüning Parache et al., 2024; Wagner et al., 2025).

Studies comparing single-mother and single-father families have generally found few consistent differences in well-being-related outcomes between these family types (Downey et al., 1998; Faulconer et al., 2022; Kleinschlömer and Krapf, 2023; Yujongdee et al., 2026). These findings suggest that children’s well-being may not differ systematically depending on whether the resident parent is the mother or the father. In the present study, single-mother and single-father families were combined into a single category of single-parent families (Ballerini et al., 2026).

However, findings from comparisons of adolescent well-being across non-intact family structures remain mixed. Some studies found no differences between single-parent and stepparent families (Schütz et al., 2019; Gordeeva et al., 2026b). Other studies reported lower levels of life satisfaction and well-being among adolescents from single-parent families than among those from stepparent families (Bjarnason et al., 2012; Ziol-Guest and Dunifon, 2014; Kuruczova et al., 2020; Grüning Parache et al., 2024), while others found the opposite pattern (Biblarz and Gottainer, 2000; Nahkur and Kutsar, 2022). This inconsistency suggests that family structure alone may not fully capture variation in adolescents’ subjective well-being and that psychological factors associated with family context should also be considered.

### 1.2 Residential care and adolescents’ subjective well-being

Children and adolescents in out-of-home care tend to report less favorable outcomes across several domains of well-being. However, methodological limitations and variation in outcome measures limit the conclusions that can be drawn. The available research suggests that adolescents in residential care report lower subjective well-being, life satisfaction, and self-esteem than adolescents who live with their parents (Dinisman et al., 2012; Golovey et al., 2017; Long et al., 2017; Nakatomi et al., 2018; Danilova and Danilova, 2020; Semya et al., 2025). They also report higher levels of depressive and anxiety symptoms (Avakyan and Volikova, 2014; Zolotareva and Khegay, 2024). Dinisman et al. (2012) showed that, for some indicators, including satisfaction with classmates, relationships with people in general, friendships, and loneliness, adolescents in care reported less favorable outcomes than those in two-parent or single-parent families. For other indicators, including happiness and overall life satisfaction, their outcomes were closer to those of adolescents in single-parent families than to those in two-parent families. In that study, the two-parent family category included both intact and stepparent families (Dinisman et al., 2012).

Recent reforms in Russia have sought to create more family-like conditions in residential care, in line with international guidelines (United Nations General Assembly, 2010). Current practice in Russian residential care institutions reflects this approach: institutions seek to maintain continuity in children’s relationships with adult caregivers who play significant roles in their lives. They house small numbers of children in mixed-age, apartment-style groups, keep siblings together, enroll children in mainstream schools outside the institution, and aim to address child’s individual needs (Semya et al., 2016, 2024). Consistent with the emphasis on family-based care, the state has also sought to increase the number of children placed with adoptive families. As a result, over the past two decades, the number of children living in residential institutions in Russia has gradually declined alongside the expansion of family-based care arrangements (Semya et al., 2024).

### 1.3 Mediators of the association between family structure and adolescents’ subjective well-being

Family income has been widely examined as a potential mediator, particularly in research addressing the lower household incomes observed among single-mother families. Low family socioeconomic status, financial hardship, and greater workloads among single mothers have been associated with fewer resources available to support children’s physical and psychological development (Ziol-Guest and Dunifon, 2014; Lin and Yi, 2019; Grüning Parache et al., 2024; Gorina et al., 2025; Hallerbäck et al., 2026). At the same time, existing research suggests that the association between family structure and children’s well-being is not fully accounted for by differences in household income and may also involve other family factors (Ziol-Guest and Dunifon, 2014; Lin and Yi, 2019). Moreover, research within self-determination theory indicates that basic psychological need satisfaction may explain a substantial proportion of the association between income and well-being, although this evidence comes from a predominantly adult sample (Martela et al., 2023).

A potential psychological mediator of this association is family relationship quality, which is closely associated with adolescents’ development and well-being (Amato, 2005; Friendly and Grolnick, 2008; Dolgikh, 2025; Yumkina et al., 2025; Gordeeva et al., 2026b; Pablos Cruz et al., 2026; Rzhanova and Alekseeva, 2026). Within self-determination theory, which provides the theoretical framework for the present study, the quality of family relationships, particularly parental autonomy support, is conceptualized as a key factor that facilitates the satisfaction of the basic psychological needs for autonomy, competence, and relatedness (Deci and Ryan, 2000; Friendly and Grolnick, 2008). In turn, greater satisfaction of these needs is associated with higher well-being and positive affect, and lower negative affect (Stanley et al., 2021). However, most of this evidence comes from adult samples and relies on general measures of need satisfaction.

Research with adolescent samples has also shown that satisfaction of basic psychological needs in relationships with parents is associated with adolescents’ life satisfaction and anxiety (Dost-Gözkan, 2021). Niemiec et al. (2006) found that high school students who perceived greater parental need support reported higher well-being, including greater life satisfaction and vitality, and lower ill-being, including fewer depressive symptoms. However, this study assessed only autonomy and relatedness support and did not report participants’ family structure (Niemiec et al., 2006). Another study with adolescents from intact families also found that parents’ basic psychological need satisfaction was associated with adolescents’ basic psychological need satisfaction and well-being (Lo Cricchio et al., 2021).

Studies examining basic psychological need satisfaction among children and adolescents across different family structures remain relatively scarce. For example, Davids et al. (2016) examined basic psychological need satisfaction and frustration as predictors of adolescents’ life goals in single-parent and two-parent families. Schrooyen et al. (2021) examined single parenthood as a potential risk factor for lower parental basic psychological need satisfaction, which was in turn associated with parents’ positive and negative feelings when spending time with their children. Recently, studies have also reported associations between basic psychological need satisfaction and well-being among adolescents living in residential care (Shouket and Dildar, 2020).

Thus, previous research suggests two broad groups of potential mediators of the association between family structure and adolescents’ well-being: economic factors, particularly family affluence, and psychological factors related to basic psychological need satisfaction within the family context. To our knowledge, no previous study has jointly examined family structure, residential care, perceived family affluence, basic psychological need satisfaction, and subjective well-being within a single SDT-based structural model. The present study therefore examines perceived family affluence and basic psychological need satisfaction at home as potential statistical mediators of the associations between family and care contexts and adolescents’ subjective well-being. Basic psychological need satisfaction at home is particularly relevant within self-determination theory, a well-established framework of motivation and well-being.

### 1.4 Theoretical background

The use of psychological theory to understand differences in children’s and adolescents’ subjective well-being across family structures remains relatively uncommon (Jensen and Sanner, 2021). The present study draws on the cultural-historical approach (Vygotsky, 1984) and self-determination theory (SDT; Deci and Ryan, 2000; Vansteenkiste et al., 2023), which provide complementary perspectives on adolescents’ development and well-being (Salikhova et al., 2021). A central concept in the cultural-historical approach is the social situation of development, which refers to the relationship between the child and the social environment in which development takes place (Vygotsky, 1984). Within this framework, relationships with significant adults are understood to be gradually internalized, becoming part of the child’s psychological functioning, with potential implications for well-being. From an SDT perspective, social environments vary in the extent to which they support the individual’s basic psychological needs for autonomy, competence, and relatedness, and greater need satisfaction is associated with higher levels of well-being, life satisfaction, and intrinsic motivation (Ryan and Deci, 2000, 2017). SDT provides a framework for examining the social situation of development in terms of the extent to which children’s basic psychological needs are supported and satisfied.

SDT identifies three basic psychological needs: autonomy, competence, and relatedness. These needs are described as fundamental psychological “nutrients” essential for growth, well-being and integrity (Deci and Ryan, 2000; Vansteenkiste et al., 2023). The need for autonomy refers to the inherent desire to act with a sense of volition and choice while experiencing self-direction in one’s actions, thoughts, and feelings. The need for competence refers to the desire to feel effective, capable and able to master challenges when interacting with the environment. The need for relatedness reflects the desire to experience warm relationships, feel connected to others, loved and unconditionally accepted. These needs are considered innate and universal across age, gender, and cultures, although the ways in which they are satisfied may vary across cultural contexts (Soenens et al., 2015).

Subjective well-being is a multidimensional psychological construct that reflects individuals’ subjective evaluations of the quality of their lives. It generally encompasses cognitive evaluations of life satisfaction and affective experiences (Diener, 1984; Diener et al., 1999). The present study adopts this conceptualization of well-being and assesses both components.

### 1.5 The present study

The present study examines the association between basic psychological need satisfaction at home and subjective well-being among adolescents living in different family structures and in residential care. Based on the preceding literature analysis, we formulate the following hypotheses.

*Hypothesis 1*: Family structure is indirectly associated with adolescents’ subjective well-being through basic psychological need satisfaction at home and through a sequential pathway involving perceived family affluence and basic psychological need satisfaction at home.

*Hypothesis 1.1*: Living in residential care is indirectly associated with adolescents’ subjective well-being through basic psychological need satisfaction at home.

*Hypothesis 2*: Adolescents from intact families have higher levels of basic psychological need satisfaction at home than adolescents from single-parent and stepparent families, while adolescents in residential care have lower levels than adolescents in each of the three family structure groups.

## 2 Materials and methods

### 2.1 Participants and sampling procedure

The participants were students in grades 5 through 9, they ranged in age from 10 to 16 years (*M*_age_ = 13.20, SD = 1.50), with a balanced gender distribution (50% girls, 50% boys). Based on their self-reported family structure, the sample was divided into four groups: intact families (*n* = 3,442; 65.0%), single-parent families (*n* = 1,123; 21.2%; 92% single-mother families), stepparent families (*n* = 628; 11.9%), and residential care (*n* = 102; 1.9%). A two-stage stratified random sampling procedure was employed. First, we randomly selected 28 secondary schools located in the vicinity of 22 residential care institutions across the Altai Krai, Russia. These comprised 14 schools in six urban settlements and 14 schools in 13 rural villages. Participants from the residential care group were distributed across all 28 schools, with cluster sizes ranging from 1 to 11 per school (*M* = 4.08, SD = 2.92). According to self-reports, their length of stay in residential care ranged from 1 to 8 years (*M* = 3.67, SD = 1.93).

The survey yielded 5,594 valid responses. Due to missing data, we were unable to classify the living arrangement of 132 adolescents (2.4%), who were therefore not included in the current research. Another 167 participants (3.0%) reported other family types that did not meet our criteria and were also excluded. The final sample consisted of 5,295 adolescents. This dataset contained no missing values on the study variables. Therefore, no imputation procedures were necessary. The perceived family affluence indicator was not administered to the 102 participants from the residential care group, as this measure is conceptually inapplicable to children living in institutional settings. Thus, missingness on this variable is structural and planned by design, rather than stochastic, and was not subjected to imputation.

Because group sizes reflected the natural distribution of participants across the studied categories rather than differential sampling, equal group sizes were neither feasible nor desirable. Consequently, *a priori* power analysis was not appropriate. Instead, the sample size (particularly *n* ≈ 100 for the smallest group) was planned to ensure sufficient precision of the group mean estimates: the standard error (σ/√N) allowed estimation of each mean within approximately ±0.2 SD (95% CI). Although unequal group sizes reduce the precision and statistical power for comparisons involving the smallest group, they do not in themselves bias between-group comparisons when the assumptions of the statistical model are satisfied. The imbalance therefore reflects the underlying population structure while preserving adequate precision for detecting medium and large effects, consistent with standard practice in psychological research.

### 2.2 Procedure

Data was collected between November 2025 and February 2026. Schools informed parents and legal guardians about the survey by email and sent a text-message notification drawing their attention to the email. Parents and legal guardians were asked to indicate whether they agreed to or declined their child’s participation. Those who agreed then provided written informed consent. The adolescents were provided with age-appropriate information about the study and were informed that participation was voluntary and that they could decline to participate or withdraw from the study at any time without any negative consequences. The students also provided assent on the first webpage of the survey by confirming that they agreed to participate in the study. Participants completed the survey anonymously.

The survey was conducted online in school classrooms using school computers and the “Testograf” online platform. All 5–9 grade students who were present at school on the day of data collection and whose parents and legal guardians had provided written informed consent were invited to participate. This study was approved by the Ethics Committee of the Faculty of Psychology at Lomonosov Moscow State University (the approval no: 2025/19) and conducted in accordance with relevant guidelines and the Declaration of Helsinki.

### 2.3 Measures

#### 2.3.1 Family structure

Family structure was assessed using a self-report item: “Please indicate who you currently live with”. Response options included mother, father, stepmother, stepfather, living in residential care, and other.

#### 2.3.2 Basic psychological need satisfaction at home scale

To assess basic psychological need satisfaction at home, we used a slightly modified Basic Psychological Need Satisfaction at School Scale (Gordeeva and Sychev, 2024) developed within the SDT framework and based on prior work (Tian et al., 2014). The questionnaire consisted of 9 items beginning with the stem “At home (where I currently live), with my parents (caregivers), I usually…”, to which adolescents responded on a 4-point Likert-type scale ranging from 1 (Not true) to 4 (True). Each subscale contained 3 items assessing satisfaction of the need for autonomy (e.g., “Feel free to make my own choices about what I do”), competence (e.g., “Feel capable and successful”), and relatedness (e.g., “Feel that my parents/caregivers are friendly and warm toward me”). A total score was computed as the mean of all items across the three subscales (English translation is added in Supplementary material). Cronbach’s α values were 0.80–0.89 for the subscales and 0.89 for the total score.

#### 2.3.3 Subjective well-being

Subjective well-being (SWB) was conceptualized as adolescents’ subjective cognitive evaluations and affective experiences of their lives. It was assessed using three indicators.

##### 2.3.3.1 Life satisfaction

To assess the cognitive component of SWB, we used an adapted version of the Brief Multidimensional Students’ Life Satisfaction Scale (BMSLSS; Huebner et al., 2006). The item pertaining to satisfaction with one’s family was excluded during analysis for all groups because it is not applicable to some participants (i.e., those in residential care), and to avoid its inflated correlations with basic psychological need satisfaction in the family, which would have arisen due to the shared specific context. The remaining 5 items (e.g., “I would describe my satisfaction with my [domain] as...”) assessed satisfaction with oneself, friends, school, teachers, and life in general on a 5-point Likert scale. The total score, computed as the mean of the 5 items, demonstrated high internal consistency (Cronbach’s *α* = 0.79).

##### 2.3.3.2 Global evaluation of life

To assess the affective component of SWB, we used a single-item non-verbal measure of global evaluation of life, the Faces Scale (Andrews and Withey, 1976). It consists of 7 schematic faces depicting varying emotional states ranging from cheerful and smiling to sad and very unhappy. Adolescents were asked to select the face that best reflected their evaluation of life in response to the prompt: “Which face best represents your attitude to life?”. Scores ranged from 1 (very negative) to 7 (very positive).

##### 2.3.3.3 Usual mood

To further assess the affective component of SWB, we used the Mood Scale which was previously employed in similar research (e.g., Odinokova et al., 2017). Adolescents were asked to respond to the question: “What is your usual mood?” on a scale from 1 (very bad) to 5 (very good).

#### 2.3.4 Perceived family affluence

Following the recommendations of Davisson et al. (2025), we used adolescents’ own subjective income evaluations rather than parent reports. Two ordinal items with response options ranging from 1 to 4 were used (Bøe et al., 2018, 2019): (1) “Which of the following best describes your family’s financial situation (choose one response): 1 — money is only enough for food; 2 — money is enough for food, but buying clothes is difficult; 3 — money is enough for food and clothes, but not for larger purchases (e.g., smartphone, computer, washing machine); 4 — money is enough for large purchases and leisure, in general for everything we need.” (2) “How would you rate your family’s level of financial security compared to others (families of your classmates)? 1 — below average, 2 — average, 3 — above average, 4 — high”. The polychoric correlation between the two items was 0.52, *p* < 0.001, Spearman-Brown reliability *r* = 0.69, which allowed us to use the sum of scores on these two items as an overall indicator of perceived family affluence.

### 2.4 Data analysis

We first generated descriptive statistics and correlations for all studied variables and assessed gender differences using Welch’s *t*-test and the Mann–Whitney *U*-test (using the R environment). Also, we estimated the effects of family structure on the studied variables using Welch’s one-way ANOVA and assessed effect sizes, using ω^2^, which provides an estimate of explained variance that is less biased than η^2^. Effect sizes were interpreted using the following cutoff values: ω^2^ < 0.01 (very small, negligible), 0.01 ≤ ω^2^ < 0.06 (small), 0.06 ≤ ω^2^ < 0.14 (medium), and ω^2^ ≥ 0.14 (large) (Kirk, 1996).

To test the hypothesis, we conducted structural equation modeling in Mplus 8.8. To account for the clustered nature of the data (adolescents nested within 28 schools), we estimated all models using cluster-robust standard errors (CLUSTER option with TYPE = COMPLEX) combined with the robust maximum likelihood estimator (MLR). All path coefficients were standardized. The coefficients for binary variables were standardized using the standard deviation of the dependent variable (STDY option in Mplus), so their values indicate the difference between the two levels of the independent variable in standard deviation units of the dependent variable. We considered the following values as indicating acceptable (good) model fit: CFI and TLI > 0.90 (0.95), and RMSEA and SRMR < 0.08 (0.06) (Mueller and Hancock, 2019). We obtained 95% bias-corrected bootstrap confidence intervals based on 5,000 resamples using the ML estimator to assess the statistical significance of indirect effects, alongside the standard estimations by the delta method. To evaluate measurement invariance, we relied on practical fit indices to compare nested models, using ΔCFI cutoff value of 0.010 as indicator of pronounced differences in fit between nested models (Cheung and Rensvold, 2002; Chen, 2007), because the chi-square difference test is known to be overly sensitive in large samples (Chen, 2007).

To assess the robustness of the results, we conducted two sensitivity analyses: a model specification analysis and an influential case analysis, examining four alternative models. In the model specification sensitivity analysis, we evaluated three alternative models: (1) a model using the full six-item BMSLSS instead of the five-item version; (2) the baseline model with correlated residuals specified between the autonomy need and relatedness need indicators in basic psychological needs factor, corresponding to the largest modification index; and (3) a model including a common method factor. The common method factor was specified to load on all indicators of the subjective well-being factor and the basic psychological need satisfaction at home factors. Its factor loadings were freely estimated, its variance was fixed at 1, and its correlations with the substantive latent factors were fixed to zero.

During the influential case analysis (alternative model 4), influential observations were identified using generalized Cook’s distance (Pek and MacCallum, 2011). The available literature does not appear to provide generally accepted cutoff values for generalized Cook’s distance. We therefore applied the interquartile range (IQR) criterion based on Tukey’s boxplot rule. This approach is implemented as the default option in the *Influence. SEM* R package (Pastore and Altoé, 2022) and has been adopted in previous research (Jaffari and Koran, 2024). Influential observations were defined as cases with generalized Cook’s distance values exceeding the upper boxplot fence (Q3 + 1.5 × IQR) of the distance distribution.

## 3 Results

### 3.1 Factor structure and measurement invariance of the questionnaires

To assess the factor structure of the Basic Psychological Need Satisfaction at Home Scale, we tested the three-factor model with freely correlated factors using CFA with the WLSMV estimator. The results demonstrated good fit: χ^2^ = 326.52; *df* = 24; *p* < 0.001; CFI = 0.996; TLI = 0.993; SRMR = 0.014; RMSEA = 0.049 (90% CI = [0.044, 0.054]); PCLOSE = 0.654; N = 5,295. The correlations among the latent factors were high (ranging from 0.73 to 0.78), supporting the examination of a higher-order general factor of basic psychological need satisfaction at home. The corresponding hierarchical model yielded equivalent fit indices, with factor loadings on the second-order factor ranging from 0.83 to 0.88. These results supported the use of an aggregated total score across the three subscales. Measurement invariance of the factor structure across current living situation, gender and age group (grade) was supported by multigroup analysis (Supplementary Table S1).

To examine the factor structure of five-item BMSLSS, we tested a unidimensional model with *a priori* specified residual covariance between the school and teacher satisfaction items, reflecting two closely related aspects of satisfaction with school life. The model showed acceptable fit to the data: χ^2^ = 109.64; *df* = 4; *p* < 0.001; CFI = 0.994; TLI = 0.986; SRMR = 0.015; RMSEA = 0.071 (90% CI = [0.060, 0.082]); PCLOSE = 0.001; *N* = 5,295. Measurement invariance of the factor structure across current living situation and age group/grade was supported by multigroup analysis (Supplementary Table S2). However, scalar invariance across gender was not supported, indicating systematic differences in item intercepts, whereas metric invariance was supported. Metric invariance permits the examination and comparison of associations involving the latent construct, such as covariances and structural paths, across gender groups. However, because scalar invariance was not established, comparisons of latent means across gender groups should be avoided and observed mean-score differences should be interpreted with caution.

### 3.2 Descriptive statistics, intercorrelations and gender differences

Descriptive statistics and correlations for all quantitative variables are presented in Table 1. As expected, indicators of adolescents’ subjective well-being were positively associated with overall basic psychological need satisfaction at home, as well as with satisfaction of each individual basic need. Older adolescents reported slightly lower levels of subjective well-being and basic psychological need satisfaction (except autonomy), although the correlations with age were small in magnitude (from −0.03 to −0.10).

**Table 1:** Descriptive statistics and Spearman correlations among measured variables.

Variable	*α*	*M*	*SD*	1	2	3	4	5	6	7	8
1. SWB: Life satisfaction	0.79	4.03	0.71	—							
2. SWB: Global evaluation of life	—	5.95	1.35	0.58^***^	—						
3. SWB: Usual mood	—	3.68	0.84	0.54^***^	0.51^***^	—					
4. BPNS at home	0.89	3.35	0.54	0.61^***^	0.51^***^	0.46^***^	—				
5. Autonomy	0.80	3.36	0.63	0.40^***^	0.35^***^	0.30^***^	0.83^***^	—			
6. Competence	0.80	3.09	0.69	0.62^***^	0.49^***^	0.48^***^	0.86^***^	0.55^***^	—		
7. Relatedness	0.89	3.60	0.61	0.48^***^	0.44^***^	0.34^***^	0.78^***^	0.57^***^	0.54^***^	—	
8. Perceived family affluence	0.69^a^	6.13	1.18	0.23^***^	0.22^***^	0.16^***^	0.27^***^	0.22^***^	0.23^***^	0.23^***^	—
9. Age	—	13.20	1.50	−0.10^***^	−0.06^***^	−0.03^*^	−0.03^*^	0.02	−0.04^**^	−0.08^***^	−0.07^***^

*p < 0.05, **p < 0.01, ***p < 0.001, α—Cronbach’s alpha (^a^ instead of Cronbach’s alpha the Spearman-Brown coefficient is presented for family affluence). No Cronbach’s α coefficient is reported for single-item scales. SWB – subjective well-being, BPNS — basic psychological need satisfaction.

Comparisons of mean scores between boys and girls are presented in Supplementary Table S3, together with the detailed results of Welch’s *t*-tests and Mann–Whitney U-tests. Boys had higher observed scores on life satisfaction [Cohen’s *d* = 0.09, *t*(5292) = 3.22, *p* = 0.001], global evaluation of life [Cohen’s *d* = 0.15, *t*(5257) = 5.54, *p* < 0.001] and usual mood [Cohen’s *d* = 0.16, *t*(5291) = 5.83, *p* < 0.001]. However, because scalar invariance was not supported for the BMSLSS across gender, the observed difference in life satisfaction should not be interpreted as evidence of a gender difference in the latent construct. Boys also showed significantly higher competence [Cohen’s *d* = 0.10, *t*(5291) = 3.54, *p* < 0.001] and relatedness need satisfaction [Cohen’s *d* = 0.06, *t*(5292) = 2.06, *p* = 0.040], but lower autonomy need satisfaction [Cohen’s *d* = 0.06, *t*(5281) = 2.18, *p* = 0.029]. The aggregated score for basic psychological need satisfaction at home did not differ significantly between boys and girls [Cohen’s *d* = 0.04, *t*(5292) = 1.43, *p* = 0.151].

Table 2 presents the means and standard deviations for the study variables across family structure groups, together with the results of Welch’s one-way ANOVAs and effect size estimates. The analyses indicated statistically significant (*p* < 0.001) but very small between-group differences in all indicators of subjective well-being: life satisfaction (ω^2^ = 0.009), global evaluation of life (ω^2^ = 0.008), and usual mood (ω^2^ = 0.005). Between-group differences in basic psychological need satisfaction at home were also statistically significant (*p* < 0.001), with an effect size of ω^2^ = 0.010.

**Table 2:** Means and standard deviations for study variables for each family structure group.

Variables	Intact family (*n* = 3,442)	Single-parent family (*n* = 1,123)	Stepparent family (*n* = 628)	Residential care (*n* = 102)	Welch’s *F* (3; *df*)	*p*(F)	ω^2^
SWB: Life satisfaction	4.08 (0.69)	3.95 (0.73)	3.94 (0.72)	3.80 (0.83)	17.73 (435.17)	<0.001	0.009
SWB: Global evaluation of life	6.04 (1.27)	5.79 (1.46)	5.76 (1.44)	5.68 (1.56)	15.35 (433.09)	<0.001	0.008
SWB: Usual mood	3.72 (0.83)	3.59 (0.83)	3.59 (0.87)	3.60 (1.00)	10.31 (435.69)	<0.001	0.005
BPNS at home	3.39 (0.52)	3.30 (0.56)	3.27 (0.61)	3.06 (0.69)	18.54 (431.30)	<0.001	0.010
Autonomy	3.38 (0.61)	3.34 (0.63)	3.31 (0.67)	3.00 (0.90)	9.10 (430.99)	<0.001	0.005
Competence	3.15 (0.67)	3.00 (0.74)	3.01 (0.73)	2.92 (0.73)	17.95 (436.31)	<0.001	0.010
Relatedness	3.64 (0.57)	3.56 (0.65)	3.51 (0.70)	3.27 (0.81)	15.10 (429.33)	<0.001	0.008

The standard deviations are presented in parentheses. Welch’s *F* — the results of Welch’s *F*-test, p(F) — statistical significance for Welch’s *F*-test, ω^2^ — effect size. SWB – subjective well-being, BPNS — basic psychological need satisfaction.

### 3.3 Assessment of the measurement model and discriminant validity

Before examining the associations between family structure, basic psychological need satisfaction at home, and subjective well-being, we evaluated the measurement model to assess the discriminant validity of the latent constructs. The measurement model included two freely correlated latent factors: a basic psychological need satisfaction at home factor indicated by the autonomy, competence, and relatedness scales, and a subjective well-being factor indicated by life satisfaction (the five-item BMSLSS), global evaluation of life (the Faces Scale), and usual mood (the Mood Scale). The model did not demonstrate acceptable overall fit: χ^2^ = 885.75, df = 8, *p* < 0.001, CFI = 0.931, TLI = 0.871, SRMR = 0.033, RMSEA = 0.144 (90% CI = [0.136, 0.152]), PCLOSE < 0.001 (Supplementary Table S4). The largest modification index corresponded to a residual covariance between the autonomy and relatedness indicators. After allowing this residual covariance, model fit improved substantially and reached an acceptable level: χ^2^ = 147.57, df = 7, *p* < 0.001, CFI = 0.989, TLI = 0.976, SRMR = 0.020, RMSEA = 0.062 (90% CI = [0.053, 0.070]), PCLOSE = 0.012 (Supplementary Table S5). Because we lacked sufficient justification for specifying a residual covariance between these indicators, we did not include this parameter in the main models. Instead, we examined a model including this residual covariance as part of the sensitivity analyses.

To further assess discriminant validity, we compared each measurement model with an otherwise identical model in which the correlation between the two latent factors was constrained to 1. In the original measurement model without correlated residuals, the constrained model fit significantly worse than the unconstrained model (∆χ^2^ = 135.48, ∆df = 1, *p* < 0.001, see Supplementary Table S6). Likewise, after allowing the residual covariance between the autonomy and relatedness indicators, the constrained model also fit significantly worse than the unconstrained model (∆χ^2^ = 114.27, ∆df = 1, *p* < 0.001, see Supplementary Table S6). The hypothesis that the correlation between the two latent factors was equal to 1 was rejected, supporting discriminant validity between the two constructs. Additional evidence was provided by the bootstrap confidence interval for the correlation between the two factors, which lay entirely below 1, with an upper bound of 0.88 (Anderson and Gerbing, 1988).

### 3.4 Analysis of relationships between family structure, perceived family affluence, basic psychological need satisfaction at home, and subjective well-being

A structural equation model was specified to test Hypothesis 1. Family structure indicators (dummy coded) were entered as predictors of perceived family affluence, basic psychological need satisfaction at home, and subjective well-being (Figure 1). Intact family status served as the reference category and was therefore not represented by a dummy variable. Accordingly, the coefficients for the single-parent and stepparent family indicators represent differences from the intact-family reference group in standard deviation units of the dependent variable. Covariances among the family structure indicators were not estimated as model parameters. Because adolescents in residential care were not asked about perceived family affluence, they were excluded from this analysis.

**Figure 1:**
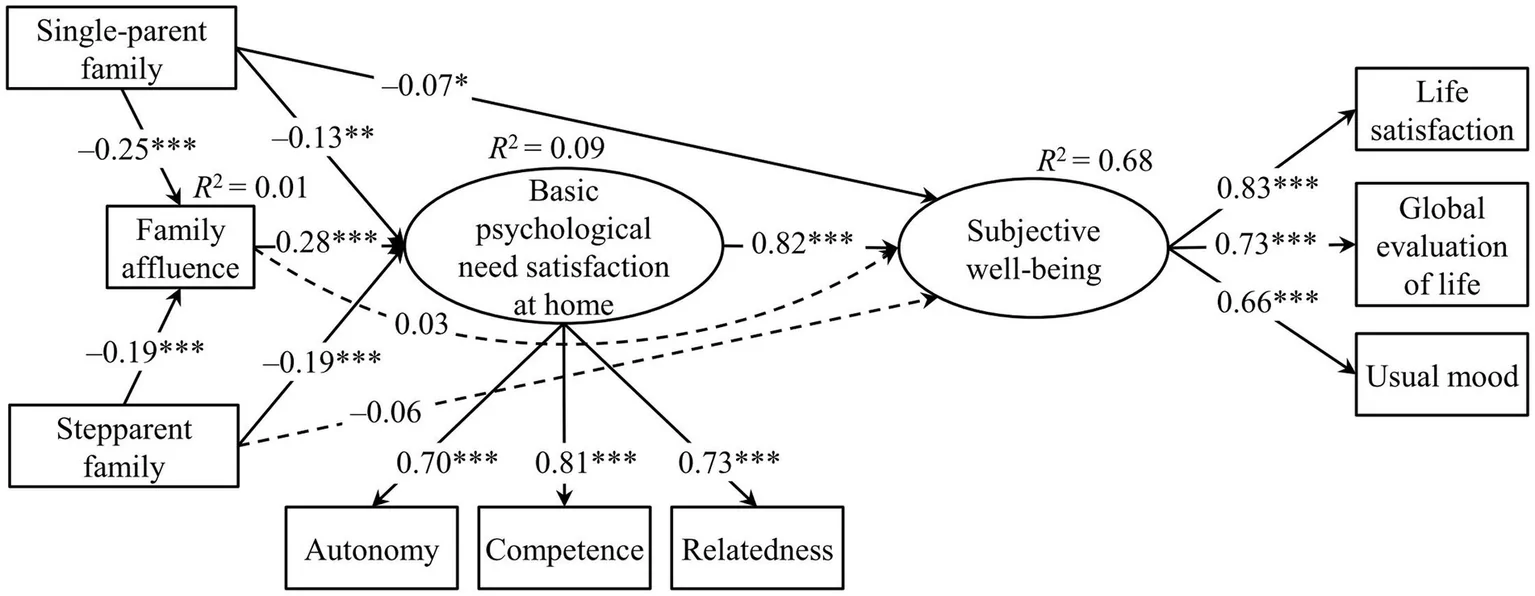
Structural model of the relationships between family structure, perceived family affluence, basic psychological need satisfaction at home, and subjective well-being. *p < 0.05, **p < 0.01, ***p < 0.001, residuals are omitted for parsimony. Total, direct and indirect effects of family structure on subjective well-being in this model are presented in Table 3.

The model (Figure 1) demonstrated acceptable overall fit to the data: χ^2^ = 411.62; *df* = 20; *p* < 0.001; CFI = 0.943; TLI = 0.900; SRMR = 0.026; RMSEA = 0.061 (90% CI = [0.056, 0.067]); PCLOSE < 0.001; *N* = 5,193. The R^2^ values were 0.68 for subjective well-being and 0.09 for basic psychological need satisfaction at home. All associations between family structure indicators and basic psychological need satisfaction at home were statistically significant (Supplementary Table S7). The direct association between single-parent family status and subjective well-being was also statistically significant. The Wald test indicated no statistically significant difference between the coefficients for single-parent and stepparent family status in their associations with basic psychological need satisfaction at home, χ^2^(1) = 0.77, *p* = 0.382. Perceived family affluence was significantly associated with basic psychological need satisfaction at home, whereas its direct association with subjective well-being was not statistically significant.

Bootstrap estimates showed that all indirect effects of family structure on subjective well-being were statistically significant. Both the indirect effects via basic psychological need satisfaction at home and the sequential indirect effects via perceived family affluence and basic psychological need satisfaction at home had 95% CIs that did not include zero (Table 3).

**Table 3:** Total, direct and indirect effects in the structural model of the relationships between family structure, perceived family affluence, basic psychological need satisfaction at home, and subjective well-being (Figure 1).

Effects	Estimate	S.E.	Est./S.E.	*p*-value	95% CI	% mediated
Effects of single-parent family						
Total	−0.25	0.05	−4.84	<0.001	[−0.35, −0.15]	
Direct	−0.07	0.04	−2.13	0.033	[−0.15, −0.01]	
Total indirect	−0.17	0.03	−5.08	<0.001	[−0.24, −0.11]	70
Via BPNS at home	−0.11	0.04	−2.50	0.013	[−0.20, −0.03]	43
Via perceived family affluence	−0.01	0.01	−0.65	0.515	[−0.03, 0.01]	3
Via perceived family affluence and BPNS at home	−0.06	0.02	−3.14	0.002	[−0.09, −0.02]	24
Effects of stepparent family						
Total	−0.26	0.05	−4.90	<0.001	[−0.36, −0.16]	
Direct	−0.06	0.05	−1.23	0.219	[−0.14, 0.04]	
Total indirect	−0.20	0.03	−6.02	<0.001	[−0.28, −0.14]	79
Via BPNS at home	−0.15	0.02	−6.45	<0.001	[−0.21, −0.11]	59
Via perceived family affluence	−0.01	0.01	−0.66	0.507	[−0.02, 0.01]	2
Via perceived family affluence and BPNS at home	−0.05	0.01	−3.25	0.001	[−0.08, −0.02]	17

Est. — Estimate, S.E. — standard error, CI — bootstrap confidence interval. BPNS — basic psychological need satisfaction.

To assess the robustness of the results, we conducted two sensitivity analyses: a model specification analysis and an influential case analysis. Excluding 588 influential observations (11%) resulted in a sample of 4,605 adolescents. All four alternative models demonstrated acceptable fit (Supplementary Table S8). All hypothesized indirect effects specified in Hypothesis 1 remained statistically significant across alternative model specifications. The indirect effect of single-parent family status on subjective well-being via basic psychological need satisfaction at home ranged from −0.13 (*p* < 0.01) to −0.09 (*p* < 0.01). The sequential indirect effect via perceived family affluence and basic psychological need satisfaction at home ranged from −0.06 (*p* < 0.01) to −0.05 (*p* < 0.001). For stepparent family status, the indirect effect via basic psychological need satisfaction at home ranged from −0.16 (*p* < 0.001) to −0.11 (*p* < 0.05), and the sequential indirect effect via perceived family affluence and basic psychological need satisfaction at home ranged from −0.07 (*p* < 0.001) to −0.04 (*p* < 0.001). The indirect effect of perceived family affluence on subjective well-being via basic psychological need satisfaction at home ranged from 0.19 (*p* < 0.001) to 0.26 (*p* < 0.001). The hypothesized indirect associations remained robust across alternative model specifications.

The direct associations of single-parent and stepparent family status with subjective well-being were small across all models, and their statistical significance varied across model specifications. The direct association between single-parent family status and subjective well-being, which was statistically significant in the baseline model, was not significant in the model with correlated residuals or in the model excluding influential observations (Supplementary Table S8). Conversely, the direct association between stepparent family status and subjective well-being was non-significant in most models but became statistically significant after influential observations were excluded. The observed instability of the direct associations between family status and subjective well-being does not contradict our hypotheses, as the hypothesized indirect associations remained statistically significant across all model specifications.

The standardized path coefficient from basic psychological need satisfaction at home to subjective well-being was 0.82 (*p* < 0.001) in the baseline model and 0.74 (*p* < 0.001) in the model including a common method factor and was therefore approximately 10% smaller in the latter model. The remaining structural path coefficients changed only slightly and retained their statistical significance. Although the model including the common method factor demonstrated the best fit to the data, which was consistent with the presence of a method-related component, the structural path coefficients changed only minimally.

Single-parent and stepparent family status were associated with lower basic psychological need satisfaction at home, which was in turn associated with lower subjective well-being, after accounting for perceived family affluence. Differences in perceived family affluence across family structures were also associated with differences in basic psychological need satisfaction at home, which were in turn associated with subjective well-being.

### 3.5 Analysis of associations between family structure, residential care, basic psychological need satisfaction at home, and subjective well-being

To test Hypothesis 1.1, we extended the model to include the residential care group and examined associations between family and care contexts, basic psychological need satisfaction at home, and subjective well-being. We expected residential care status to be associated with lower basic psychological need satisfaction at home than the family structure categories. Because perceived family affluence was not assessed in the residential care group, it was not included in this model.

The model (Figure 2) demonstrated acceptable fit to the data: χ^2^ = 565.27; *df* = 20; *p* < 0.001; CFI = 0.996; TLI = 0.993; SRMR = 0.027; RMSEA = 0.072 (90% CI = [0.067, 0.077]); PCLOSE < 0.001; *N* = 5,295. The *R*^2^ values were 0.69 for subjective well-being and 0.02 for basic psychological need satisfaction at home. All coefficients linking the family and care indicators to basic psychological need satisfaction at home were statistically significant, as was the direct association between single-parent family status and subjective well-being (Supplementary Table S9). Bootstrap estimation showed that all indirect effects of the family and care indicators on subjective well-being via basic psychological need satisfaction at home were statistically significant (Table 4).

**Figure 2:**
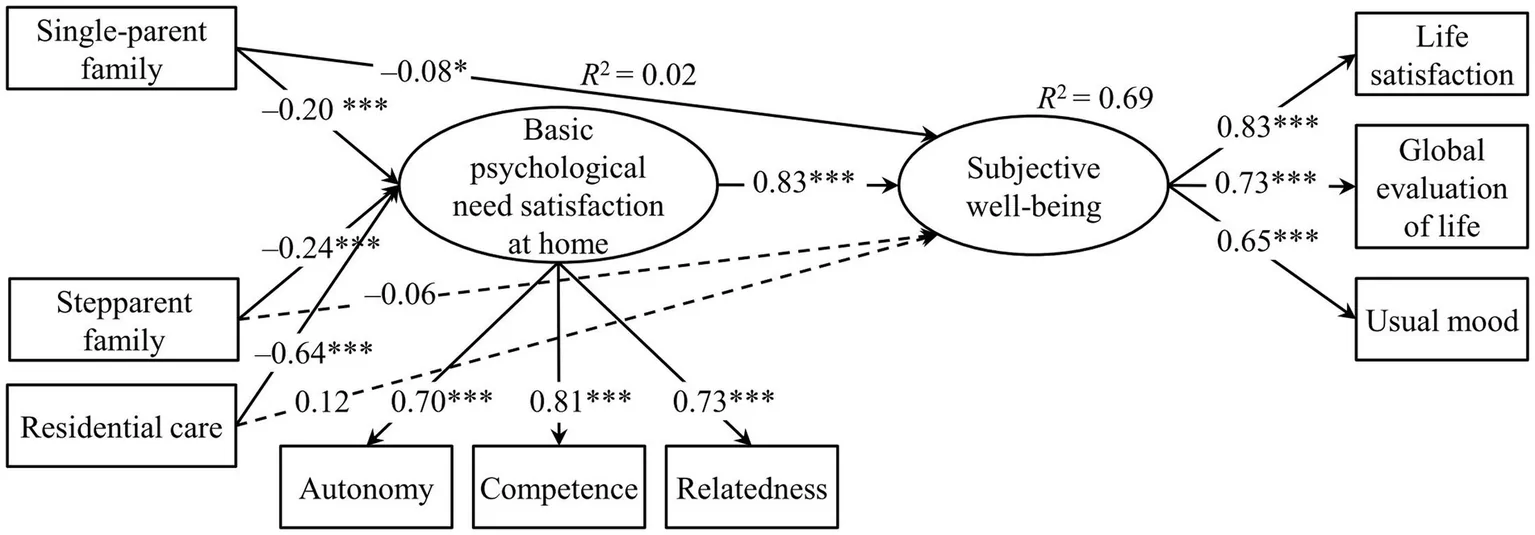
Structural model of the relationships between family structure, residential care, basic psychological need satisfaction at home, and subjective well-being. *p < 0.05, **p < 0.01, ***p < 0.001, residuals are omitted for parsimony. Total, direct and indirect effects of family structure on subjective well-being in this model are presented in Table 4.

**Table 4:** Total, direct and indirect effects in the structural model of the relationships between family structure, residential care, basic psychological need satisfaction at home, and subjective well-being (Figure 2).

Effects	Estimate	S. E.	Est./S. E.	p-value	95% CI	% mediated
Effects of single-parent family						
Total	−0.25	0.05	−4.84	< 0.001	[−0.35, −0.15]	
Direct	−0.08	0.04	−2.30	0.022	[−0.15, −0.02]	
Indirect via BPNS at home	−0.17	0.04	−4.74	< 0.001	[−0.24, −0.10]	67
Effects of stepparent family						
Total	−0.26	0.05	−4.88	< 0.001	[−0.36, −0.16]	
Direct	−0.06	0.05	−1.29	0.198	[−0.15, 0.04]	
Indirect	−0.20	0.03	−6.29	< 0.001	[−0.27, −0.14]	77
Effects of residential care						
Total	−0.41	0.14	−2.88	0.004	[−0.68, −0.12]	
Direct	0.12	0.10	1.13	0.259	[−0.17, 0.32]	
Indirect via BPNS at home	−0.53	0.13	−4.22	< 0.001	[−0.77, −0.28]	n.a.^a^

^a^ In this case the total effect was smaller than the indirect effect, so the correct estimate of percentage mediated is not available. Est.—Estimate, S.E.—standard error, CI—bootstrap confidence interval. BPNS—basic psychological need satisfaction.

To test Hypothesis 2, we used a Wald test to examine whether the coefficients linking the family and care indicators to basic psychological need satisfaction at home differed in magnitude. The omnibus Wald test was statistically significant, χ^2^(2) = 7.37, *p* = 0.025. Bonferroni-adjusted pairwise comparisons showed that the coefficient for residential care differed significantly from those for single-parent and stepparent family status (*p* < 0.05).

To assess the robustness of the results, we conducted two sensitivity analyses: a model specification analysis and an influential case analysis. Excluding 667 influential observations (13%) resulted in a sample of 4,628 adolescents. All alternative models demonstrated acceptable fit (Supplementary Table S10). The model including a common method factor could not be estimated under the clustering specification because the number of free parameters (31) exceeded the number of clusters (28).

All hypothesized indirect effects remained statistically significant across alternative model specifications. The indirect association between residential care and subjective well-being via basic psychological need satisfaction at home ranged from −0.67 (*p* < 0.001) to −0.47 (*p* < 0.001). The corresponding indirect association for stepparent family status ranged from −0.20 (*p* < 0.001) to −0.17 (*p* < 0.001). The largest change in magnitude was observed for the indirect association between single-parent family status and subjective well-being via basic psychological need satisfaction at home, which changed from −0.17 (*p* < 0.001) in the baseline model to −0.09 (*p* < 0.05) after influential observations were excluded, while remaining statistically significant. The hypothesized indirect associations between family and care contexts and adolescents’ subjective well-being therefore remained robust across alternative model specifications.

The direct associations of residential care and stepparent family status with subjective well-being were small and non-significant across all model specifications. The direct association between single-parent family status and subjective well-being was statistically significant in the baseline model (−0.08, *p* < 0.05) but was not statistically significant in the model with correlated residuals (−0.06).

Wald tests of equality constraints on the paths from the family and care indicators to basic psychological need satisfaction at home were statistically significant across all model specifications except the model with correlated residuals, in which the omnibus Wald test was not statistically significant, χ^2^(2) = 5.27, *p* = 0.072. Evidence for differences in the magnitude of these associations was therefore present in most, but not all, alternative model specifications.

The results showed significant and robust indirect associations between family and care contexts and subjective well-being through basic psychological need satisfaction at home. The observed pattern was consistent with the hypothesized indirect association through basic psychological need satisfaction at home. In the baseline model, adolescents from intact families reported the highest levels of basic psychological need satisfaction at home, adolescents from single-parent and stepparent families reported lower levels, and adolescents in residential care reported the lowest levels. However, differences in the magnitude of these associations were not fully robust across alternative model specifications.

## 4 Discussion

The present study examined the association between satisfaction of basic psychological needs at home (i.e., autonomy, competence, and relatedness) and subjective well-being among Russian adolescents living in different types of families and care. The findings showed significant indirect associations between living in different family structures or in residential care and adolescents’ subjective well-being through basic psychological need satisfaction at home. These findings suggest that satisfaction of basic psychological needs at home varies across family and care contexts associated with differences in subjective well-being.

Hypothesis 1 was supported. Family structure was indirectly associated with adolescents’ subjective well-being through basic psychological need satisfaction at home and through a sequential pathway involving perceived family affluence and basic psychological need satisfaction at home. These indirect associations remained statistically significant across the sensitivity analyses. In contrast, the direct associations of single-parent and stepparent family status with subjective well-being were small and unstable across model specifications, with their statistical significance changing in the sensitivity analyses. No firm conclusion can therefore be drawn about residual direct associations in these models and further research is needed on this issue.

These findings are consistent with previous research within SDT showing that basic psychological need satisfaction in the family context is associated with adolescents’ well-being (Dost-Gözkan, 2021; Lo Cricchio et al., 2021). This study also extends previous SDT research by examining basic psychological need satisfaction at home across different family structures.

Our findings indicate that perceived family affluence was indirectly associated with subjective well-being through basic psychological need satisfaction at home, whereas its direct association with subjective well-being was not statistically significant. This finding is consistent with evidence from adolescent samples (Yoo and Choi, 2016; Zhang et al., 2021), as well as from adult samples across several European countries (González et al., 2016; Martela et al., 2023). The present findings are also consistent with research suggesting that family income does not fully account for associations between family structure and indicators of well-being and mental health (Ziol-Guest and Dunifon, 2014; Grüning Parache et al., 2024), as well as with Zaborskis et al. (2022), who reported a stronger association of family support than family affluence with adolescents’ life satisfaction.

Hypothesis 1.1 was supported. Living in residential care was indirectly associated with adolescents’ subjective well-being through basic psychological need satisfaction at home, and this indirect association remained statistically significant across the sensitivity analyses. This finding extends the pattern observed across different family structures to adolescents living in residential care. The comparatively low levels of basic psychological need satisfaction at home reported by adolescents in residential care are discussed in more detail later in this section. Although the cross-sectional design does not allow causal conclusions about mediation, the observed model is consistent with SDT and suggests that basic psychological need satisfaction at home is an important psychological factor associated with adolescents’ subjective well-being across family and care contexts.

Hypothesis 2 was supported in the baseline model. Adolescents from intact families reported higher levels of basic psychological need satisfaction at home than adolescents from single-parent and stepparent families, whereas adolescents in residential care reported lower levels than adolescents in all three family structure groups. Thus, adolescents from single-parent and stepparent families occupied an intermediate position between those from intact families and those living in residential care. However, differences in the magnitude of these associations were not fully robust across alternative model specifications, and the observed ordering should therefore be interpreted with caution.

The finding that adolescents from intact families reported greater satisfaction of their basic psychological needs at home was consistent with expectations and helps explain their higher levels of subjective well-being. Several characteristics of two-parent families identified in previous research may help account for this pattern, such as parents’ own life satisfaction and basic psychological need satisfaction (Duineveld et al., 2017; Lo Cricchio et al., 2021), lower parental stress (McDonnell and Gracia, 2024), fewer financial difficulties, and higher parental educational attainment (McDonnell and Gracia, 2024; Gorina et al., 2025).

Our findings are consistent with previous studies reporting no differences in subjective well-being between adolescents from single-parent and stepparent families (Schütz et al., 2019; Gordeeva et al., 2026b). The results on absence of differences in overall basic psychological need satisfaction at home between these two groups are consistent with this pattern. The similarity between these two groups suggests that adolescents’ well-being may be associated not simply with the number of adults with whom they live, but also with the quality of their relationships, as potentially reflected in basic psychological need satisfaction at home. Despite the absence of differences between adolescents from single-parent and stepparent families, adolescents in these family structures may experience different sources of stress and distinct challenges. In stepparent families, household resources and biological parents’ life satisfaction may increase (Dierker et al., 2025). At the same time, children may need to adapt to changes in family relationships and develop a relationship with a new parental figure, a process that may involve conflict (Ganong and Sanner, 2023; Gold and Edin, 2023). By contrast, single parents are substantially more likely than parents in stable partnerships to experience financial hardship (McDonnell and Gracia, 2024; Gorina et al., 2025; Ballerini et al., 2026; Hallerbäck et al., 2026), social isolation and limited social support (McDonnell and Gracia, 2024; Hallerbäck et al., 2026), greater responsibility for childcare, and work–family conflict (McDonnell and Gracia, 2024) that may place additional strain on parenting.

Adolescents in residential care reported lower overall basic psychological need satisfaction at home than adolescents living in family settings. This pattern is consistent with previous research emphasizing the importance of supportive relationships with adults in residential care (Huynh et al., 2019). This finding is also consistent with Shouket and Dildar (2020), who found a significant association between basic psychological need satisfaction and life satisfaction among adolescents in residential care. Lower basic psychological need satisfaction may reflect both adverse early experiences in the family of origin and adolescents’ current social situation of development within residential care. Parental alcohol dependence, for example, has been documented in Russian reports as a common reason for placement in residential care (Analytical Center for the Government of the Russian Federation, 2019). Although residential care in many countries, including Russia, has increasingly been organized in more family-like settings, frequent caregiver turnover and high child-to-caregiver ratios remain common in residential care settings (Hermenau et al., 2017; Wright et al., 2019).

The present findings suggest that basic psychological need satisfaction at home is an important psychological factor linking family structure and living in residential care to adolescents’ subjective well-being. The observed associations indicate that differences in subjective well-being may be related not only to characteristics such as family structure, living in residential care, and family affluence, but also to a significant extent to adolescents’ experience of family environment, namely the extent to which their basic psychological needs are satisfied and met at home. In terms of practical implications, parent and caregiver education focused on supporting adolescents’ basic psychological needs in autonomy, competence, and relatedness may be a useful component of psychological assistance for adolescents growing up in vulnerable family and care contexts.

## 5 Strengths and limitations

This study has several chief strengths. First, to our knowledge, it is the first study to simultaneously examine basic psychological need satisfaction at home across four family and care contexts among adolescents. Second, the use of a large sample of Russian adolescents and structural equation modeling allowed us to examine the associations among family structure, perceived family affluence, basic psychological need satisfaction at home, and adolescents’ subjective well-being. Third, the study moved beyond comparisons between intact families and a single combined group of non-intact families. It examined differences across distinct family and care contexts, namely intact families, single-parent families, stepparent families, and residential care, in adolescents’ basic psychological need satisfaction at home and subjective well-being.

The present study is not without limitations. First, the cross-sectional design does not allow for causal inferences. Cross-sectional questionnaire data can identify associations among variables but cannot establish causality (Taris et al., 2021). Nevertheless, our empirical findings are consistent with theoretical propositions regarding the relationships among these constructs (Vygotsky, 1984; Ryan and Deci, 2000). Second, the study lacked information about when the family became a single-parent or stepparent family, which made it impossible to determine whether adolescents had lived in a single-parent family from the outset or how much time had passed since parental separation, divorce, or death. For example, previous research on single-mother families suggests that long-term life satisfaction may depend on whether the child lived with the mother throughout childhood and adolescence or only during part of this period (Richter and Lemola, 2017), as well as on whether the father was absent from the outset or died (Biblarz and Gottainer, 2000). At the study design stage, we made a deliberate decision not to ask adolescents detailed questions about family transitions to avoid potential distress, in line with ethical considerations. Third, the residential care group was relatively small, which indicates the need to replicate the findings in larger samples. Fourth, the study relied on adolescents’ self-report data, which allowed us to examine their subjective perceptions of the family environment, basic psychological need satisfaction, and subjective well-being. Reliance on a single source of data, particularly self-report measures of basic psychological need satisfaction and subjective well-being, may introduce common method bias and socially desirable responding. However, in the sensitivity analysis including a common method factor, the association between basic psychological need satisfaction and subjective well-being was approximately 10% smaller but remained statistically significant, and all hypothesized indirect associations involving family structure remained statistically significant. Thus, the findings remained unchanged after accounting for a common method factor.

## 6 Conclusion

The findings extend current understanding of the associations between family structure and care context and adolescents’ subjective well-being by showing consistent with theory significant statistical mediation through basic psychological need satisfaction at home. Perceived family affluence was also only indirectly associated with adolescents’ subjective well-being through basic psychological need satisfaction at home, whereas its direct association with subjective well-being was not statistically significant. Adolescents from intact families reported higher basic psychological need satisfaction at home than those from single-parent and stepparent families, while adolescents in residential care reported the lowest levels. These findings highlight that supporting adolescents’ basic psychological needs at home may be an important focus for parents and caregivers across diverse family and care contexts, particularly for adolescents growing up in socially and economically vulnerable circumstances.

## Data Availability

The datasets presented in this study can be found in online repositories (https://osf.io/evxmu/).
