# Supplementary material for: Family structure, family affluence and adolescents’ subjective well-being: the mediating role of basic psychological need satisfaction
Source: Front Psychol. 2026 Oct 29;17:1958295. doi: 10.3389/fpsyg.2026.1958295 (PMC13755289; doi:10.3389/fpsyg.2026.1958295)
Supplement: Supplementary file 1 [file Supplementary_file_1.docx]

Supplementary Material

# 1 Supplementary Data

## Basic Psychological Need Satisfaction at Home Scale (Translation from Russian into English)

Please read each statement carefully and choose the response that best describes your experience. Use the following scale: *Not true (NO)*, *Somewhat not true*, *Somewhat true*, *True (YES)*.

**At home (where I currently live), with my parents(caregivers), I usually…**

| **Statement** | **Not true (NO)** | **Somewhat not true** | **Somewhat true** | **True (YES)** |
| --- | --- | --- | --- | --- |
| 1. Feel free to make my own choices about what I do. |  |  |  |  |
| 2. Do well with my homework. |  |  |  |  |
| 3. Feel supported by my parents/caregivers. |  |  |  |  |
| 4. Can choose what things to do and what activities to take part in. |  |  |  |  |
| 5. Feel that I am good at the things I do. |  |  |  |  |
| 6. Feel that my parents/caregivers are friendly and warm toward me. |  |  |  |  |
| 7. Have many opportunities to do things I want to do and that are important to me. |  |  |  |  |
| 8. Feel capable and successful. |  |  |  |  |
| 9. Feel that my parents/caregivers are ready to help me. |  |  |  |  |

**Subscales:**

**Autonomy:** Items 1, 4, 7

**Competence:** Items 2, 5, 8

**Relatedness:** Items 3, 6, 9

# 2 Supplementary Tables

## Supplementary Table S1

*Measurement invariance testing of the basic psychological need satisfaction scale at home scale*

| Model | χ^2^ | *df* | *p* | CFI | TLI | SRMR | RMSEA | ΔCFI |
| --- | --- | --- | --- | --- | --- | --- | --- | --- |
| *Across different family structure groups* | | | | | | | | |
| 1. Configural | 380.07 | 96 | < 0.001 | 0.996 | 0.994 | 0.015 | 0.047 |  |
| 2. Measurement | 365.38 | 114 | < 0.001 | 0.996 | 0.995 | 0.016 | 0.041 | < 0.001 |
| 3. Scalar | 399.29 | 159 | < 0.001 | 0.996 | 0.997 | 0.017 | 0.034 | < 0.001 |
| *Across gender groups* | | | | | | | | |
| 1. Configural | 377.79 | 48 | < 0.001 | 0.995 | 0.993 | 0.015 | 0.051 |  |
| 2. Measurement | 362.11 | 54 | < 0.001 | 0.996 | 0.994 | 0.016 | 0.046 | 0.001 |
| 3. Scalar | 412.52 | 69 | < 0.001 | 0.995 | 0.995 | 0.017 | 0.043 | –0.001 |
| *Across age groups/grades* | | | | | | | | |
| 1. Configural | 418.66 | 120 | < 0.001 | 0.996 | 0.994 | 0.016 | 0.049 |  |
| 2. Measurement | 391.92 | 144 | < 0.001 | 0.997 | 0.996 | 0.017 | 0.041 | 0.001 |
| 3. Scalar | 494.00 | 204 | < 0.001 | 0.996 | 0.996 | 0.019 | 0.037 | –0.001 |

## Supplementary Table S2

*Measurement invariance testing of the five–item BMSLSS*

| Model | χ^2^ | *df* | *p* | CFI | TLI | SRMR | RMSEA | ΔCFI |
| --- | --- | --- | --- | --- | --- | --- | --- | --- |
| *Across different family structure groups* | | | | | | | | |
| 1. Configural | 132.73 | 16 | < 0.001 | 0.993 | 0.983 | 0.016 | 0.074 |  |
| 2. Measurement | 106.16 | 28 | < 0.001 | 0.995 | 0.994 | 0.016 | 0.046 | 0.002 |
| 3. Scalar | 187.63 | 70 | < 0.001 | 0.993 | 0.996 | 0.020 | 0.036 | –0.002 |
| *Across gender groups* | | | | | | | | |
| 1. Configural | 100.57 | 8 | < 0.001 | 0.995 | 0.988 | 0.014 | 0.066 |  |
| 2. Measurement | 96.49 | 12 | < 0.001 | 0.996 | 0.993 | 0.015 | 0.052 | 0.001 |
| 3. Scalar | 324.35 | 26 | < 0.001 | 0.984 | 0.988 | 0.023 | 0.066 | –0.012 |
| *Across age groups/grades* | | | | | | | | |
| 1. Configural | 130.23 | 20 | < 0.001 | 0.994 | 0.985 | 0.017 | 0.073 |  |
| 2. Measurement | 114.21 | 36 | < 0.001 | 0.996 | 0.994 | 0.018 | 0.046 | 0.002 |
| 3. Scalar | 274.29 | 92 | < 0.001 | 0.990 | 0.995 | 0.024 | 0.044 | –0.006 |

## Supplementary Table S3

*Comparison of boys and girls on study variables*

| Variables | Mean (F) | Mean (M) | SD (F) | SD (M) | Welch’s t | *df* | *p(t)* | Cohen's *d* | U | *p*(U) |
| --- | --- | --- | --- | --- | --- | --- | --- | --- | --- | --- |
| SWB: Life satisfaction | 4.00 | 4.06 | 0.71 | 0.71 | –3.22 | 5292 | 0.001 | –0.09 | 3313049 | 0.001 |
| SWB: Global evaluation of life | 5.85 | 6.05 | 1.41 | 1.28 | –5.54 | 5257 | < 0.001 | –0.15 | 3206323 | < 0.001 |
| SWB: Usual mood | 3.61 | 3.74 | 0.83 | 0.84 | –5.83 | 5291 | < 0.001 | –0.16 | 3164442 | < 0.001 |
| BPNS at home | 3.34 | 3.36 | 0.54 | 0.54 | –1.43 | 5292 | 0.151 | –0.04 | 3416433 | 0.112 |
| Autonomy need | 3.38 | 3.34 | 0.62 | 0.64 | 2.18 | 5281 | 0.029 | 0.06 | 3612978 | 0.045 |
| Competence need | 3.06 | 3.13 | 0.70 | 0.68 | –3.54 | 5291 | < 0.001 | –0.10 | 3319040 | < 0.001 |
| Relatedness need | 3.58 | 3.62 | 0.62 | 0.61 | –2.06 | 5292 | 0.040 | –0.06 | 3389325 | 0.020 |

*Note.* F — female, M — male. U — Mann-Whitney test. SWB – subjective well-being, BPNS — basic psychological need satisfaction.

## Supplementary Table S4

*The* *results of measurement model analysis* (Fit indexes: χ^2^ = 885.75; df = 8; p < 0.001; CFI = 0.931; TLI = 0.871; SRMR = 0.033; RMSEA = 0.144 (90% CI = [0.136, 0.152]); PCLOSE < 0.001; N = 5295)

| **Parameter 1** | **Command** | **Parameter 2** | **Estimate** | **S.E.** | **Est./S.E** | ***p*** | **95% CI** |
| --- | --- | --- | --- | --- | --- | --- | --- |
| BPNS at home | BY | Autonomy | 0.70 | 0.02 | 30.68 | < 0.001 | [0.65, 0.74] |
| BPNS at home | BY | Competence | 0.81 | 0.02 | 42.76 | < 0.001 | [0.78, 0.85] |
| BPNS at home | BY | Relatedness | 0.73 | 0.02 | 45.81 | < 0.001 | [0.70, 0.76] |
| SWB | BY | Satisfaction with life | 0.83 | 0.02 | 46.36 | < 0.001 | [0.79, 0.86] |
| SWB | BY | Global evaluation of life | 0.73 | 0.02 | 49.91 | < 0.001 | [0.69, 0.75] |
| SWB | BY | Usual mood | 0.65 | 0.02 | 28.01 | < 0.001 | [0.61, 0.70] |
| SWB | WITH | BPNS at home | 0.83 | 0.02 | 38.09 | < 0.001 | [0.79, 0.87] |
| Intercepts |  | Global evaluation of life | 4.41 | 0.16 | 27.33 | < 0.001 | [4.13, 4.79] |
| Intercepts |  | Usual mood | 4.38 | 0.07 | 67.07 | < 0.001 | [4.24, 4.50] |
| Intercepts |  | Autonomy | 5.34 | 0.16 | 32.86 | < 0.001 | [5.04, 5.69] |
| Intercepts |  | Competence | 4.46 | 0.10 | 43.16 | < 0.001 | [4.26, 4.67] |
| Intercepts |  | Relatedness | 5.88 | 0.27 | 22.21 | < 0.001 | [5.41, 6.46] |
| Intercepts |  | Satisfaction with life | 5.70 | 0.12 | 45.99 | < 0.001 | [5.47, 5.95] |
| Variances |  | BPNS at home | 1.00 | 0.00 | — | — | — |
| Variances |  | SWB | 1.00 | 0.00 | — | — | — |
| Residual variances |  | Global evaluation of life | 0.47 | 0.02 | 22.39 | < 0.001 | [0.44, 0.52] |
| Residual variances |  | Usual mood | 0.57 | 0.03 | 18.81 | < 0.001 | [0.51, 0.63] |
| Residual variances |  | Autonomy | 0.51 | 0.03 | 16.08 | < 0.001 | [0.45, 0.57] |
| Residual variances |  | Competence | 0.34 | 0.03 | 11.13 | < 0.001 | [0.28, 0.40] |
| Residual variances |  | Relatedness | 0.47 | 0.02 | 20.18 | < 0.001 | [0.42, 0.51] |
| Residual variances |  | Satisfaction with life | 0.32 | 0.03 | 10.70 | < 0.001 | [0.26, 0.37] |

*Note.* BPNS at home — basic psychological need satisfaction at home, SWB — subjective well-being.

## Supplementary Table S5

*The* *results of analysis for modified measurement model* (Fit indexes: χ^2^ = 147.57; df = 7; p < 0.001; CFI = 0.989; TLI = 0.976; SRMR = 0.020; RMSEA = 0.062 (90% CI = [0.053, 0.070]); PCLOSE = 0.012; N = 5295)

| **Parameter 1** | **Command** | **Parameter 2** | **Estimate** | **S.E.** | **Est./S.E** | ***p*** | **95% CI** |
| --- | --- | --- | --- | --- | --- | --- | --- |
| BPNS at home | BY | Autonomy | 0.63 | 0.02 | 31.97 | < 0.001 | [0.60, 0.67] |
| BPNS at home | BY | Competence | 0.85 | 0.02 | 56.37 | < 0.001 | [0.83, 0.88] |
| BPNS at home | BY | Relatedness | 0.66 | 0.01 | 47.15 | < 0.001 | [0.63, 0.69] |
| SWB | BY | Satisfaction with life | 0.83 | 0.02 | 48.12 | < 0.001 | [0.80, 0.86] |
| SWB | BY | Global evaluation of life | 0.72 | 0.02 | 47.47 | < 0.001 | [0.69, 0.75] |
| SWB | BY | Usual mood | 0.66 | 0.02 | 28.30 | < 0.001 | [0.62, 0.70] |
| SWB | WITH | BPNS at home | 0.85 | 0.02 | 47.07 | < 0.001 | [0.81, 0.88] |
| Relatedness | WITH | Autonomy | 0.30 | 0.02 | 14.11 | < 0.001 | [0.26, 0.34] |
| Intercepts |  | Global evaluation of life | 4.41 | 0.16 | 27.33 | < 0.001 | [4.13, 4.79] |
| Intercepts |  | Usual mood | 4.38 | 0.07 | 67.07 | < 0.001 | [4.24, 4.50] |
| Intercepts |  | Autonomy | 5.34 | 0.16 | 32.86 | < 0.001 | [5.04, 5.69] |
| Intercepts |  | Competence | 4.46 | 0.10 | 43.16 | < 0.001 | [4.26, 4.67] |
| Intercepts |  | Relatedness | 5.88 | 0.27 | 22.21 | < 0.001 | [5.41, 6.46] |
| Intercepts |  | Satisfaction with life | 5.70 | 0.12 | 45.99 | < 0.001 | [5.47, 5.95] |
| Variances |  | BPNS at home | 1.00 | 0.00 | — | — | — |
| Variances |  | SWB | 1.00 | 0.00 | — | — | — |
| Residual variances |  | Global evaluation of life | 0.48 | 0.02 | 22.18 | < 0.001 | [0.44, 0.53] |
| Residual variances |  | Usual mood | 0.57 | 0.03 | 18.63 | < 0.001 | [0.51, 0.62] |
| Residual variances |  | Autonomy | 0.60 | 0.03 | 23.79 | < 0.001 | [0.55, 0.64] |
| Residual variances |  | Competence | 0.27 | 0.03 | 10.58 | < 0.001 | [0.22, 0.32] |
| Residual variances |  | Relatedness | 0.56 | 0.02 | 30.09 | < 0.001 | [0.52, 0.60] |
| Residual variances |  | Satisfaction with life | 0.31 | 0.03 | 10.83 | < 0.001 | [0.25, 0.36] |

*Note.* BPNS at home — basic psychological need satisfaction at home, SWB — subjective well-being.

## Supplementary Table S6

*Comparison results for the base and restricted measurement models*

| **Model number and type** | **χ^2^** | ***df*** | **CFI** | **TLI** | **RMSEA** | **SRMR** | **SCF** | **Comparison** | **∆χ^2^** | **∆df** | **p** |
| --- | --- | --- | --- | --- | --- | --- | --- | --- | --- | --- | --- |
| 1. Base measurement model | 885.75 | 8 | 0.931 | 0.871 | 0.144 | 0.033 | 0.600 | – | – | – | – |
| 2. Restricted measurement model (SWB WITH BPNS at home is fixed at 1) | 1104.72 | 9 | 0.914 | 0.857 | 0.152 | 0.051 | 1.037 | model 1  vs. model 2 | 135.48 | 1 | < 0.001 |
| 3. Base measurement model + correlated errors for autonomy and relatedness | 147.57 | 7 | 0.989 | 0.976 | 0.062 | 0.020 | 1.465 | – | – | – | – |
| 4. Restricted measurement model + correlated errors for autonomy and relatedness (SWB WITH BPNS at home is fixed at 1) | 344.62 | 8 | 0.974 | 0.951 | 0.089 | 0.036 | 1.678 | model 3  vs. model 4 | 114.27 | 1 | < 0.001 |

*Note.* SCF — Scaling Correction Factor for MLR, ∆χ^2^ — corrected χ^2^ difference, BPNS at home — basic psychological need satisfaction at home, SWB — subjective well-being.

## Supplementary Table S7

*STDY estimates of parameters in the structural model of the relationships between family structure, perceived family affluence, basic psychological need satisfaction at home, and subjective well-being (see Figure 1 in the article)*

| **Parameter 1** | **Command** | **Parameter 2** | **Estimate** | **S.E.** | **Est./S.E** | **p** | **95% CI** |
| --- | --- | --- | --- | --- | --- | --- | --- |
| BPNS at home | BY | Autonomy | 0.70 | 0.02 | 30.77 | < 0.001 | [0.65, 0.74] |
| BPNS at home | BY | Competence | 0.81 | 0.02 | 46.48 | < 0.001 | [0.78, 0.85] |
| BPNS at home | BY | Relatedness | 0.73 | 0.02 | 47.79 | < 0.001 | [0.70, 0.76] |
| SWB | BY | Satisfaction with life | 0.83 | 0.02 | 49.17 | < 0.001 | [0.80, 0.86] |
| SWB | BY | Global evaluation of life | 0.73 | 0.01 | 51.42 | < 0.001 | [0.70, 0.75] |
| SWB | BY | Usual mood | 0.66 | 0.02 | 29.60 | < 0.001 | [0.62, 0.70] |
| SWB | ON | BPNS at home | 0.82 | 0.03 | 26.60 | < 0.001 | [0.76, 0.87] |
| BPNS at home | ON | Perceived family affluence | 0.28 | 0.07 | 3.98 | < 0.001 | [0.12, 0.38] |
| BPNS at home | ON | Single-parent family | –0.13 | 0.05 | –2.58 | 0.010 | [–0.23, –0.04] |
| BPNS at home | ON | Stepparent family | –0.19 | 0.03 | –6.42 | < 0.001 | [–0.25, –0.13] |
| SWB | ON | Single-parent family | –0.07 | 0.03 | –2.18 | 0.029 | [–0.15, –0.01] |
| SWB | ON | Stepparent family | –0.06 | 0.05 | –1.24 | 0.215 | [–0.14, 0.04] |
| SWB | ON | Perceived family affluence | 0.03 | 0.04 | 0.67 | 0.501 | [–0.05, 0.09] |
| AFFLUENC | ON | Single-parent family | –0.25 | 0.04 | –6.86 | < 0.001 | [–0.32, –0.18] |
| AFFLUENC | ON | Stepparent family | –0.19 | 0.05 | –4.24 | < 0.001 | [–0.28, –0.10] |
| Intercepts |  | Global evaluation of life | 3.50 | 0.45 | 7.77 | < 0.001 | [2.86, 4.47] |
| Intercepts |  | Usual mood | 3.56 | 0.32 | 11.17 | < 0.001 | [3.11, 4.24] |
| Intercepts |  | Autonomy | 4.44 | 0.40 | 11.21 | < 0.001 | [3.84, 5.26] |
| Intercepts |  | Competence | 3.32 | 0.36 | 9.33 | < 0.001 | [2.83, 4.11] |
| Intercepts |  | Relatedness | 4.92 | 0.47 | 10.45 | < 0.001 | [4.20, 5.96] |
| Intercepts |  | Perceived family affluence | 5.25 | 0.26 | 20.37 | < 0.001 | [4.66, 5.63] |
| Intercepts |  | Satisfaction with life | 4.68 | 0.43 | 10.89 | < 0.001 | [4.06, 5.57] |
| Residual variances |  | Global evaluation of life | 0.47 | 0.02 | 22.71 | < 0.001 | [0.43, 0.51] |
| Residual variances |  | Usual mood | 0.57 | 0.03 | 19.33 | < 0.001 | [0.51, 0.62] |
| Residual variances |  | Autonomy | 0.52 | 0.03 | 16.39 | < 0.001 | [0.46, 0.58] |
| Residual variances |  | Competence | 0.34 | 0.03 | 12.14 | < 0.001 | [0.29, 0.39] |
| Residual variances |  | Relatedness | 0.47 | 0.02 | 20.89 | < 0.001 | [0.42, 0.51] |
| Residual variances |  | Perceived family affluence | 0.99 | 0.00 | 315.70 | < 0.001 | [0.98, 0.99] |
| Residual variances |  | Satisfaction with life | 0.31 | 0.03 | 11.21 | < 0.001 | [0.26, 0.36] |
| Residual variances |  | BPNS at home | 0.91 | 0.04 | 22.64 | < 0.001 | [0.85, 0.98] |
| Residual variances |  | SWB | 0.32 | 0.03 | 9.21 | < 0.001 | [0.25, 0.38] |

*Note.* BPNS at home — basic psychological need satisfaction at home, SWB — subjective well-being.

## Supplementary Table S8

*The* *results of sensitivity analysis for the model of the relationships between family structure, perceived family affluence, basic psychological need satisfaction at home, and subjective well-being*

| **Parameter** | **Models** | | | | |
| --- | --- | --- | --- | --- | --- |
|  | Baseline model | Alternative model 1 | Alternative model 2 | Alternative model 3 | Alternative model 4 |
| **Direct effects** | | | | | |
| BPNS at home on perceived family affluence | 0.28***  [0.12, 0.38] | 0.28***  [0.12, 0.38] | 0.28***  [0.10, 0.38] | 0.26***  [0.13, 0.35] | 0.30***  [0.13, 0.40] |
| BPNS at home on single parent family | –0.13**  [–0.23, –0.04] | –0.13*  [–0.23, –0.04] | –0.15**  [–0.26, –0.07] | –0.13**  [–0.22, –0.05] | –0.13*  [–0.27, –0.05] |
| BPNS at home on stepparent family | –0.19***  [–0.25, –0.13] | –0.19***  [–0.25, –0.14] | –0.19***  [–0.26, –0.13] | –0.19***  [–0.25, –0.14] | –0.16***  [–0.25, –0.03] |
| SWB on BPNS at home | 0.82***  [0.76, 0.87] | 0.82***  [0.77, 0.88] | 0.84***  [0.79, 0.88] | 0.74***  [0.69, 0.80] | 0.85***  [0.82, 0.90] |
| SWB on family affluence | 0.03  [–0.05, 0.09] | 0.04  [–0.04, 0.10] | 0.03  [–0.03, 0.08] | 0.07  [–0.01, 0.14] | 0.01  [–0.07, 0.05] |
| SWB on single parent family | –0.07*  [–0.15, –0.01] | –0.08*  [–0.15, –0.01] | –0.05  [–0.12, 0.01] | –0.07*  [–0.15, –0.01] | –0.03  [–0.08, 0.04] |
| SWB on stepparent family | –0.06  [–0.14, 0.04] | –0.06  [–0.15, 0.04] | –0.05  [–0.13, 0.04] | –0.07  [–0.15, 0.02] | –0.10*  [–0.25, –0.06] |
| **Indirect effects** | | | | | |
| BPNS at home on single parent family via perceived family affluence | –0.07**  [–0.11, –0.02] | –0.07**  [–0.11, –0.02] | –0.07**  [–0.11, –0.02] | –0.07**  [–0.10, –0.03] | –0.06**  [–0.11, –0.02] |
| BPNS at home on stepparent family via perceived family affluence | –0.06**  [–0.10, –0.02] | –0.06**  [–0.10, –0.03] | –0.05**  [–0.10, –0.02] | –0.05**  [–0.09, –0.02] | –0.08**  [–0.12, –0.03] |
| SWB on family affluence via BPNS at home | 0.23***  [0.10, 0.3] | 0.23***  [0.11, 0.3] | 0.23***  [0.08, 0.31] | 0.19***  [0.10, 0.25] | 0.26***  [0.11, 0.35] |
| SWB on single parent family via BPNS at home | –0.11*  [–0.20, –0.03] | –0.11*  [–0.20, –0.03] | –0.13**  [–0.22, –0.05] | –0.09**  [–0.17, –0.04] | –0.13*  [–0.24, –0.04] |
| SWB on single parent family via perceived family affluence | –0.01  [–0.03, 0.01] | –0.01  [–0.03, 0.01] | –0.01  [–0.02, 0.01] | –0.02  [–0.04, 0.00] | 0.00  [–0.01, 0.01] |
| SWB on single parent family via perceived family affluence and BPNS at home | –0.06**  [–0.09, –0.02] | –0.06**  [–0.09, –0.02] | –0.06**  [–0.09, –0.02] | –0.05***  [–0.08, –0.02] | –0.06**  [–0.09, –0.02] |
| SWB on stepparent family via BPNS at home | –0.15***  [–0.21, –0.11] | –0.16***  [–0.21, –0.11] | –0.16***  [–0.22, –0.11] | –0.14***  [–0.19, –0.10] | –0.11*  [–0.22, –0.02] |
| SWB on stepparent family via perceived family affluence | –0.01  [–0.02, 0.01] | –0.01  [–0.02, 0.01] | –0.01  [–0.02, 0.01] | –0.01  [–0.03, 0.00] | 0.00  [–0.01, 0.02] |
| SWB on stepparent family via perceived family affluence and BPNS at home | –0.05***  [–0.08, –0.02] | –0.05***  [–0.08, –0.02] | –0.05**  [–0.08, –0.02] | –0.04***  [–0.06, –0.02] | –0.07***  [–0.11, –0.03] |
| **Fit indexes** | | | | | |
| χ^2^ | 411.62 | 381.25 | 178.23 | 46.94 | 342.51 |
| df | 20 | 20 | 19 | 14 | 20 |
| p | < 0.001 | < 0.001 | < 0.001 | < 0.001 | < 0.001 |
| CFI | 0.943 | 0.947 | 0.977 | 0.995 | 0.947 |
| TLI | 0.900 | 0.908 | 0.957 | 0.988 | 0.907 |
| SRMR | 0.026 | 0.025 | 0.018 | 0.011 | 0.026 |
| RMSEA | 0.061 | 0.059 | 0.040 | 0.021 | 0.059 |
| 95% CI for RMSEA | [0.056, 0.067] | [0.054, 0.064] | [0.035, 0.046] | [0.015, 0.028] | [0.054, 0.065] |

*Note.* Alternative models: (1) the model with the full six-item BMSLSS instead of the five-item version; (2) the baseline model with correlated residuals for the autonomy and relatedness indicators; (3) the model with a common method factor; (4) the baseline model after excluding influential observations (in this model *N* = 4,605, 588 (11%) cases were excluded). BPNS — basic psychological need satisfaction, SWB — subjective well-being. * — *p* < 0,05, ** — *p* < 0,01, *** — *p* < 0,001.

## Supplementary Table S9

*STDY estimates of parameters in the structural model of the relationships between family structure, residential care, basic psychological need satisfaction at home, and subjective well-being (see Figure 2 in the article)*

| **Parameter 1** | **Command** | **Parameter 2** | **Estimate** | **S.E.** | **Est./S.E** | **p** | **95% CI** |
| --- | --- | --- | --- | --- | --- | --- | --- |
| BPNS at home | BY | Autonomy | 0.70 | 0.02 | 30.80 | < 0.001 | [0.66, 0.74] |
| BPNS at home | BY | Competence | 0.81 | 0.02 | 42.05 | < 0.001 | [0.77, 0.85] |
| BPNS at home | BY | Relatedness | 0.73 | 0.02 | 45.80 | < 0.001 | [0.70, 0.76] |
| SWB | BY | Satisfaction with life | 0.83 | 0.02 | 46.48 | < 0.001 | [0.79, 0.86] |
| SWB | BY | Global evaluation of life | 0.73 | 0.02 | 49.90 | < 0.001 | [0.70, 0.75] |
| SWB | BY | Usual mood | 0.65 | 0.02 | 27.95 | < 0.001 | [0.61, 0.70] |
| SWB | ON | BPNS at home | 0.83 | 0.02 | 37.13 | < 0.001 | [0.78, 0.87] |
| SWB | ON | Single-parent family | –0.08 | 0.03 | –2.35 | 0.019 | [–0.15, –0.02] |
| SWB | ON | Stepparent family | –0.06 | 0.05 | –1.30 | 0.194 | [–0.15, 0.04] |
| SWB | ON | Residential care | 0.12 | 0.10 | 1.17 | 0.242 | [–0.10, 0.32] |
| BPNS at home | ON | Single-parent family | –0.20 | 0.04 | –5.06 | < 0.001 | [–0.28, –0.13] |
| BPNS at home | ON | Stepparent family | –0.24 | 0.04 | –6.17 | < 0.001 | [–0.33, –0.17] |
| BPNS at home | ON | Residential care | –0.64 | 0.16 | –4.13 | < 0.001 | [–0.93, –0.33] |
| Intercepts |  | Autonomy | 5.40 | 0.16 | 33.57 | < 0.001 | [5.10, 5.74] |
| Intercepts |  | Competence | 4.52 | 0.10 | 43.41 | < 0.001 | [4.33, 4.74] |
| Intercepts |  | Relatedness | 5.94 | 0.26 | 22.52 | < 0.001 | [5.47, 6.52] |
| Intercepts |  | Global evaluation of life | 4.48 | 0.16 | 27.85 | < 0.001 | [4.20, 4.85] |
| Intercepts |  | Usual mood | 4.44 | 0.07 | 67.68 | < 0.001 | [4.29, 4.56] |
| Intercepts |  | Satisfaction with life | 5.78 | 0.12 | 46.67 | < 0.001 | [5.54, 6.02] |
| Residual variances |  | Autonomy | 0.51 | 0.03 | 16.05 | < 0.001 | [0.45, 0.57] |
| Residual variances |  | Competence | 0.35 | 0.03 | 11.08 | < 0.001 | [0.28, 0.40] |
| Residual variances |  | Relatedness | 0.47 | 0.02 | 20.00 | < 0.001 | [0.42, 0.51] |
| Residual variances |  | Global evaluation of life | 0.47 | 0.02 | 22.31 | < 0.001 | [0.43, 0.52] |
| Residual variances |  | Usual mood | 0.57 | 0.03 | 18.76 | < 0.001 | [0.51, 0.63] |
| Residual variances |  | Satisfaction with life | 0.32 | 0.03 | 10.77 | < 0.001 | [0.26, 0.37] |
| Residual variances |  | BPNS at home | 0.98 | 0.00 | 247.04 | < 0.001 | [0.97, 0.99] |
| Residual variances |  | SWB | 0.31 | 0.04 | 8.68 | < 0.001 | [0.25, 0.38] |

*Note.* BPNS at home — basic psychological need satisfaction at home, SWB — subjective well-being.

## Supplementary Table S10

*The* *results of sensitivity analysis for the model of the relationships between family structure, residential care, basic psychological need satisfaction at home, and subjective well-being*

| **Parameter** | **Models** | | | |
| --- | --- | --- | --- | --- |
|  | Baseline model | Alternative model 1 | Alternative model 2 | Alternative model 3 |
| **Direct effects** | | | | |
| BPNS at home on single parent family | –0.20*** [–0.28, –0.13] | –0.20*** [–0.28, –0.12] | –0.22*** [–0.30, –0.15] | –0.10* [–0.20, –0.01] |
| BPNS at home on stepparent family | –0.24*** [–0.33, –0.17] | –0.24*** [–0.33, –0.17] | –0.24*** [–0.34, –0.16] | –0.19*** [–0.30, –0.09] |
| BPNS at home on residential care | –0.64*** [–0.93, –0.33] | –0.65*** [–0.94, –0.34] | –0.55*** [–0.84, –0.27] | –0.77*** [–0.97, –0.61] |
| SWB on BPNS at home | 0.83*** [0.78, 0.87] | 0.84*** [0.80, 0.88] | 0.85*** [0.81, 0.88] | 0.86*** [0.82, 0.90] |
| SWB on single parent family | –0.08* [–0.15, –0.02] | –0.08* [–0.16, –0.02] | –0.06 [–0.12, 0.00] | –0.08** [–0.14, –0.02] |
| SWB on stepparent family | –0.06 [–0.15, 0.04] | –0.07 [–0.15, 0.03] | –0.05 [–0.13, 0.04] | –0.06 [–0.13, 0.01] |
| SWB on residential care | 0.12 [–0.10, 0.32] | 0.05 [–0.16, 0.27] | 0.06 [–0.17, 0.27] | 0.00 [–0.24, 0.19] |
| **Indirect effects** |  |  |  |  |
| SWB on single parent family via BPNS at home | –0.17*** [–0.24, –0.10] | –0.17*** [–0.24, –0.10] | –0.19*** [–0.26, –0.13] | –0.09* [–0.18, –0.01] |
| SWB on stepparent family via BPNS at home | –0.20*** [–0.27, –0.14] | –0.20*** [–0.27, –0.15] | –0.20*** [–0.28, –0.14] | –0.17*** [–0.27, –0.08] |
| SWB on residential care via BPNS at home | –0.53*** [–0.77, –0.28] | –0.54*** [–0.79, –0.29] | –0.47*** [–0.71, –0.24] | –0.67*** [–0.86, –0.51] |
| **Fit indexes** | | | | |
| χ^2^ | 565.27 | 535.17 | 191.93 | 542.29 |
| df | 20 | 20 | 19 | 20 |
| p | < 0.001 | < 0.001 | < 0.001 | < 0.001 |
| CFI | 0.996 | 0.994 | 0.999 | 0.998 |
| TLI | 0.993 | 0.990 | 0.998 | 0.996 |
| SRMR | 0.027 | 0.027 | 0.019 | 0.030 |
| RMSEA | 0.072 | 0.070 | 0.041 | 0.075 |
| 95% CI for RMSEA | [0.067, 0.077] | [0.065, 0.075] | [0.036, 0.047] | [0.070, 0.081] |

*Note.* Alternative models: (1) the model with the full six-item BMSLSS instead of the five-item version; (2) the baseline model with correlated residuals for the autonomy and relatedness indicators; (3) the baseline model after excluding influential observations (in this model *N* = 4,628, 667 (13%) cases were excluded). BPNS — basic psychological need satisfaction, SWB — subjective well-being. * — *p* < 0,05, ** — *p* < 0,01, *** — *p* < 0,001.
